# Clinical Characteristics of Asymptomatic and Symptomatic Pediatric Coronavirus Disease 2019 (COVID-19): A Systematic Review

**DOI:** 10.3390/medicina56090474

**Published:** 2020-09-15

**Authors:** Sojung Yoon, Han Li, Keum Hwa Lee, Sung Hwi Hong, Dohoon Kim, Hyunjoon Im, Woongchan Rah, Eunseol Kim, Seungyeon Cha, Jinho Yang, Andreas Kronbichler, Daniela Kresse, Ai Koyanagi, Louis Jacob, Ramy Abou Ghayda, Jae Il Shin, Lee Smith

**Affiliations:** 1 College of Medicine, Yonsei University, Seoul 03722, Korea; sj4068@yonsei.ac.kr (S.Y.); sunghwihong@gmail.com (S.H.H.); cpt2011@nate.com (D.K.); hjoon_im@yonsei.ac.kr (H.I.); wcrah@yonsei.ac.kr (W.R.); eunseolkim@yonsei.ac.kr (E.K.); kiara486@naver.com (S.C.); yangkidoki@gmail.com (J.Y.); 2 College of Medicine, University of Florida, Gainesville, FL 32610, USA; lih2@ufl.edu; 3 Department of Pediatrics, College of Medicine, Yonsei University, Seoul 03722, Korea; AZSAGM@yuhs.ac; 4 Department of Global Health and Population, Harvard T.H. Chan School of Public Health, Boston, MA 02115, USA; ramy.aboughayda@gmail.com; 5 Department of Internal Medicine IV (Nephrology and Hypertension), Medical University Innsbruck, 6020 Innsbruck, Austria; Andreas.Kronbichler@i-med.ac.at; 6 Department of Internal Medicine, St. Johann County Hospital, 6380 St. Johann, Austria; danielakresse92@gmail.com; 7 Research and Development Unit, Parc Sanitari Sant Joan de Déu, CIBERSAM, 08830 Barcelona, Spain; a.koyanagi@pssjd.org (A.K.); louis.jacob.contacts@gmail.com (L.J.); 8 ICREA, Pg. Lluis Companys 23, 08010 Barcelona, Spain; 9 Faculty of Medicine, University of Versailles Saint-Quentin-en-Yvelines, 78000 Versailles, France; 10 Division of Urology, Brigham and Women’s Hospital, and Harvard Medical School, Boston, MA 02115, USA; 11 The Cambridge Centre for Sport and Exercise Science, Anglia Ruskin University, Cambridge CB1 1PT, UK; Lee.Smith@anglia.ac.uk

**Keywords:** coronavirus disease 2019 (COVID-19), novel coronavirus 2019, SARS-CoV-2, asymptomatic, child, infant, characteristics, radiography

## Abstract

*Background and objectives:* Characterization of pediatric coronavirus disease 2019 (COVID-19) is necessary to control the pandemic, as asymptomatic or mildly infected children may act as carriers. To date, there are limited reports describing differences in clinical, laboratory, and radiological characteristics between asymptomatic and symptomatic infection, and between younger and older pediatric patients. The objective of this study is to compare characteristics among: (1) asymptomatic versus symptomatic and (2) less than 10 versus greater or equal to 10 years old pediatric COVID-19 patients. *Materials and Methods:* We searched for all terms related to pediatric COVID-19 in electronic databases (Embase, Medline, PubMed, and Web of Science) for articles from January 2020. This protocol followed the Preferred Reporting Items for Systematic Reviews and Meta-Analysis guidelines. *Results:* Eligible study designs included case reports and series, while we excluded comments/letters, reviews, and literature not written in English. Initially, 817 articles were identified. Forty-three articles encompassing 158 confirmed pediatric COVID-19 cases were included in the final analyses. Lymphocytosis and high CRP were associated with symptomatic infection. Abnormal chest CT more accurately detected asymptomatic COVID-19 in older patients than in younger ones, but clinical characteristics were similar between older and younger patients. *Conclusions:* Chest CT scan findings are untrustworthy in younger children with COVID-19 as compared with clinical findings, or significant differences in findings between asymptomatic to symptomatic children. Further studies evaluating pediatric COVID-19 could contribute to potential therapeutic interventions and preventive strategies to limit spreading.

## 1. Introduction

Since December 2019, the severe acute respiratory syndrome coronavirus 2 (SARS-CoV-2) which causes coronavirus disease 2019 (COVID-19) emerged as a virus causing substantial morbidity and mortality worldwide. There have been over 10 million confirmed cases and over 500,000 deaths worldwide [1]. The transmission of COVID-19 has been shown to occur during pre-symptomatic or asymptomatic periods which contribute to widespread transmission of the infection [2].

The reported number of cases worldwide implicates that elderly persons are especially vulnerable to the disease, contracting from children [3,4]. In addition, most children with COVID-19 have been reported to present with mild symptoms [5]. However, many studies suggested that children, despite mild or no symptoms, could be a source of viral transmission [6,7]. It has been reported that the viral load in asymptomatic patients was similar to that in symptomatic patients, especially during the early phase of infection [8,9].

There have been some systematic reviews and meta-analyses on COVID-19 aimed at characterizing SARS-CoV-2 infections in pediatric age groups [10]. However, no study, to date, has focused on the difference between symptomatic and asymptomatic pediatric patients. Previous studies were mainly based on information from adult populations and limited data were available for children with COVID-19 [5,11,12]. Therefore, we conducted a systematic review with the aim to investigate clinical, laboratory, and radiographic characteristics and differences among symptomatic and asymptomatic COVID-19 confirmed children.

## 2. Methods

### 2.1. Goals

The primary goal of this study was to systematically evaluate and characterize reported pediatric COVID-19 cases. An age threshold of 10 years was selected to distinguish between findings before and after the onset of puberty, which can vary with ethnicity and gender but generally begins as early as age 10. Analyses were stratified by clinical characteristics, laboratory findings, and radiological signs in children with COVID-19 based on this age threshold of 10 years and the presence or absence of symptoms. For asymptomatic patients, the continued absence of symptoms was also evaluated when reported at follow-up.

### 2.2. Search Strategy

The following databases were utilized: Embase, Medline, PubMed, and Web of Science, using the search terms: “COVID-19”, “SARS-CoV-2”, “novel coronavirus 2019”, “pediatrics”, “child*”, “infant*”, “neonate*”, and “adolescent*”. Articles were evaluated independently by four different researchers (D. Kim, H. Im, W. Rha, and E. Kim). The protocol followed the recommendations set forth by the Preferred Reporting Items for Systematic Reviews and Meta-Analyses (PRISMA) statement [13].

### 2.3. Eligible Criteria

We included peer-reviewed articles that reported confirmed SARS-CoV-2 infections by reverse transcription polymerase chain reaction. Eligible study designs included case reports and case series. Comments/letters and reviews were excluded. Articles not published in English were also excluded. Articles with duplicate cases were excluded.

### 2.4. Study Selection and Assessment of Risk of Bias

Initial results were reviewed by screening for duplicates, and then were screened by title and format for eligibility criteria. After full-text screening, a targeted search was conducted to generate additional articles focusing on asymptomatic children with COVID-19, using the same method used for the initial search.

### 2.5. Data Extraction and Statistical Analysis

Three independent investigators extracted data from each eligible study using a standardized data extraction form. Data were cross-checked for duplicate articles, duplicate cases, or discrepancies. Disagreements between reviewers regarding the data were resolved with reviews from three other independent investigators (E. Kim, S. Cha, and J. Yang). Statistical analyses were performed using SPSS version 26.0. Power was calculated using G*Power version 3.1.9.6. Percentages and means ± standard deviation were used to summarize categorical and continuous variables, respectively. The Kolmogorov–Smirnov test was used to test the normal distribution and the Mann–Whitney *U* test was used for non-normal distribution variables. The Chi-square test was performed to evaluate differences between age groups and symptomatic/asymptomatic groups, while the number in each cell was below 5, Fisher’s exact test was performed, and the student’s T test was used to evaluate differences in age.

## 3. Results

**Figure 1:**
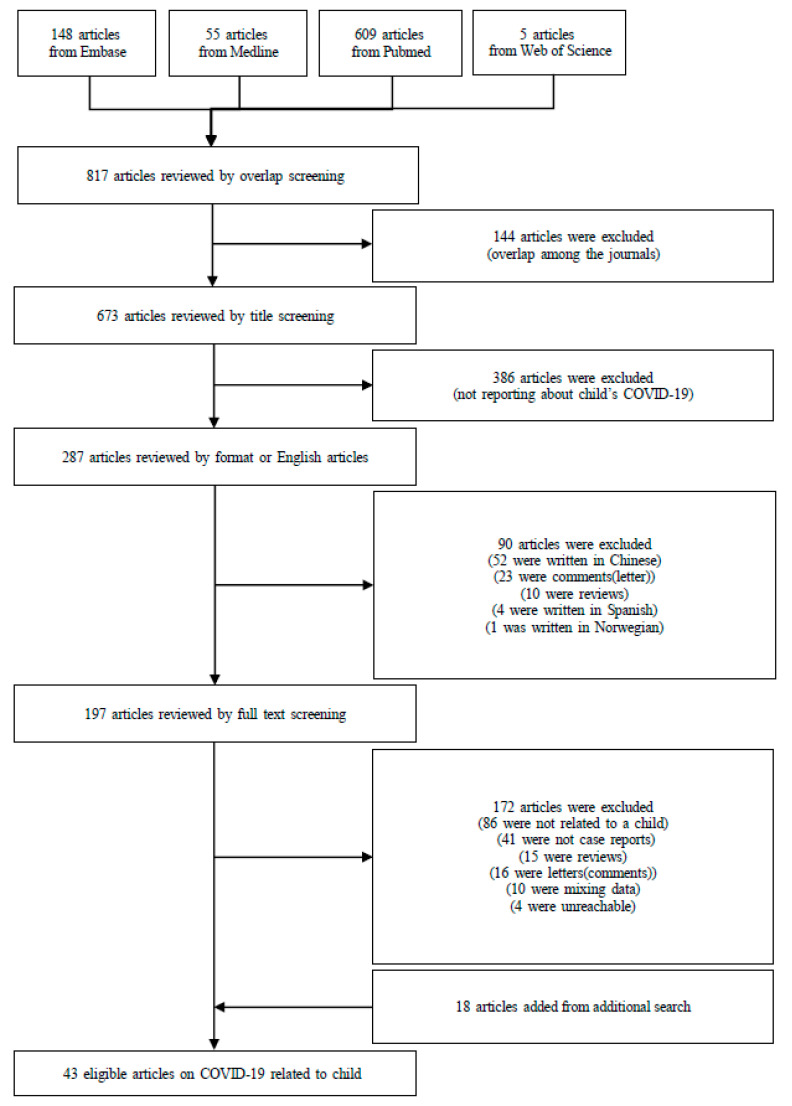
Flow chart of the literature search. Abbreviation: COVID-19 (coronavirus disease 2019).

Initially, 817 articles were identified in a preliminary search. After screening to exclude duplicates, 673 articles were reviewed by title screening for topic (focusing on pediatric COVID-19), format (excluding comments and reviews), and language. Of 197 reviewed from full-text screening, 86 were unrelated to pediatric COVID-19, 41 were not case reports, 31 were letters or reviews, 10 were pooled patient data, and 4 were inaccessible. An additional search was conducted and resulted in 18 additional eligible articles. Forty-three articles met all inclusion criteria and were included in final analyses (Figure 1).

### 3.1. Study Characteristics and Demographic Features

Of the 33 studies that reported study location, 22 took place in China [6,14,15,16,17,18,19,20,21,22,23,24,25,26,27,28,29,30,31,32,33], five in Europe [34,35,36,37,38], two in the United States [39,40], and one each in Malaysia [41], Republic of Korea [42], Vietnam [43], and Singapore [44] (Table 1). A total of 158 cases of pediatric COVID-19 were identified. All articles reported age at onset of infection. Specifically, 116 children were younger than 10 years at onset, and 40 were 10–17 years old. Twenty-four studies reported 51 asymptomatic infections (Table S1) [6,14,15,16,17,18,19,20,22,30,32,33,34,41,44,45,46,47,48,49,50,51,52], and 30 studies reported 106 cases of symptomatic infections (Table S2) [15,16,17,18,19,20,21,23,24,25,26,27,28,29,30,31,32,35,36,37,38,39,40,41,42,43,45,46,52,53]. Fever was recorded in 78 patients, respiratory symptoms (such as cough, tachypnea, or pharyngeal congestion) in 74 patients, and gastrointestinal symptoms (including vomiting and diarrhea) in 27 patients. Other symptoms included malaise, convulsions, arthralgia, headache, chest pain, fatigue, skin rash, feeding difficulty, decreased oral intake, drowsiness, and myalgia. Within the symptomatic population, 81 children were below 10 years old, and 25 were between 10–17 years old. Within the asymptomatic population, 35 were younger than 10. Sex was reported in 150 cases, and out of these 81 (54.0%) were male. In the 51 asymptomatic patients, follow-up data were reported in 24 cases (47.0%). 

### 3.2. Clinical Characteristics Based on the Presence of Symptoms

Table 1 summarizes the characteristics of all symptomatic and asymptomatic patients, and Table 2 stratifies these two groups based on their ages (<10 or ≥10 years). Twenty-three out of 24 asymptomatic patients with follow-up remained asymptomatic throughout the time course of infection. Symptomatic patients were more likely to be female (*p* = 0.054). Additionally, low creatinine levels were reported in 10 out of 83 (12.0%) symptomatic patients and none of the asymptomatic patients (*p* = 0.060). High C-reactive protein (CRP) levels were reported in 21 of 92 (22.8%) symptomatic patients and only two out of 35 (5.7%) asymptomatic cases (*p* = 0.037). Symptomatic cases appeared more likely to have a high lymphocyte count (14.0% symptomatic vs. 0% asymptomatic, *p* = 0.019) and high procalcitonin levels (54.5% symptomatic vs. 12.5% asymptomatic), although the latter was only of borderline significance (*p* = 0.092). 

Within children <10 years, one abnormal laboratory characteristic was observed more frequently in the symptomatic population, i.e., high lymphocyte counts (0.0% vs. 15.9%, *p* = 0.032, Table 2). Abnormal radiological findings, such as typical ground-glass opacities only (0.0% vs. 15.7%, *p* = 0.049) and ground-glass opacities with consolidation (8.3% vs. 0%, *p* = 0.099), occurred more often in the symptomatic group but were not significant. Within children ≥10 years, abnormal laboratory findings distinguished asymptomatic and symptomatic populations with borderline significance (*p* = 0.056).

### 3.3. Clinical Characteristics Based on Age

Table 1 summarizes the characteristics of patients according to age (i.e., ≥10 years or <10 years), and Table 3 characterizes these two groups based on the presence of symptoms. The mean age for patients below 10 years old was 3.09 years, and the mean age for patients at or above 10 was 12.60 years (Table 1). Older patients were more likely to have an increased serum creatinine (*p* = 0.065), although this was not significant. Children above 10 years of age were more likely to present with GGOs (32.0% <10 years vs. 56.5% ≥10 years, *p* = 0.049). 

Stratified by the presence of symptoms, among asymptomatic carriers, children above or equal to 10 years were more likely to present with abnormal radiological findings. Five out of six children (83%) above 10 years demonstrated typical ground-glass opacities with accompanying radiological signs of pneumonia. These signs were present in 20.8% of children below 10 years (*p* = 0.009). Likewise, among symptomatic cases of pediatric COVID-19, those of 10 years and above more frequently showed increased creatinine levels (*p* = 0.063). 

### 3.4. Treatment and Outcomes

No deaths were reported among symptomatic and asymptomatic populations. ICU admission was reported in 16 of 40 cases in the symptomatic population, and 5 of 18 cases in the asymptomatic group (Supplementary Materials Tables S1 and S2). Two were neonates delivered from SARS-CoV-2 infected women and immediately transferred to neonatal ICU (NICU) for isolation and observation. The reasons for ICU admission for the other three children were not reported. All 25 asymptomatic cases with outcome data were discharged, improved, or stable following treatment. Out of 15 neonatal cases, six cases were asymptomatic. Seven asymptomatic patients received antivirals or immunoglobulin therapy. Three of 41 symptomatic cases with outcome data remained in the ICU at the studies’ conclusion, whereas the rest were stable or recovered. Most recovered following antiviral, antibiotic, and/or oxygen therapy. Seven recovered without the need of supportive care. One patient with a history of sickle cell disease developed acute respiratory distress syndrome (ARDS) in the symptomatic group, who recovered following non-invasive ventilation, red blood cell exchange and simple transfusion, anticoagulation therapy, and one pulse of intravenous tocilizumab [36]. 

## 4. Discussion

With the rapid and continued propagation of SARS-CoV-2 infection, it is critical that its mode of transmission and the clinical characteristics of infection are clearly delineated. The pediatric population has recently been examined by numerous systematic reviews [5,10,54], both to establish clinical characteristics of pediatric infection and to evaluate the extent that children may act as asymptomatic or mildly symptomatic carriers of the disease. Although it is suspected that mildly symptomatic pediatric cases can become rapid transmitters of SARS-CoV-2 during the incubation period [55], we were unable to find studies identifying predisposing risk factors or clinical characteristics distinguishing symptomatic from asymptomatic patients. Furthermore, although many case studies were comprised of either infants or adolescents, no study, to the best of our knowledge, separated COVID-19 pediatric clinical features into younger and older age categories. Therefore, our study serves to (1) identify distinguishing features of symptomatic from asymptomatic infection and (2) characterize COVID-19 in young (<10 years) and older (10–17 years) pediatric populations. Forty-three articles published up to 4 September 2020 were aggregated. Twenty-two were conducted in China, five in Europe, two in the USA, and one each in Malaysia, the Republic of Korea, Vietnam, and Singapore. Twenty-three out of 24 asymptomatic patients remained asymptomatic throughout follow-up, decreasing the possibility that symptomatic patients experiencing an incubation period were falsely included in the asymptomatic group.

This systematic review reveals that symptomatic infection more frequently presents with abnormal laboratory characteristics indicative of infection. Low creatinine levels and high CRP, indicators of abnormal renal function and inflammatory response, respectively, were present at higher rates in symptomatic cases. Diagnostic markers more accurately determined the onset of symptoms in the population below 10 years of age. Within this group, abnormal laboratory characteristics, especially high lymphocyte levels, were associated with symptomatic infections, and abnormal radiological findings, low WBC count, low neutrophil count, and low creatinine levels neared statistical significance. This finding builds on works by Tian et al. [56], and Shi et al. [57] which linked serum creatinine and CRP to COVID-19 mortality, suggesting these markers also coincide with symptoms in pediatric patients. Interestingly, male sex was associated with asymptomatic infection. Previous studies in adults identified male sex as being associated with more severe infection, and a female predominance in asymptomatic infections [58,59,60]. Dong et al. extensively examined a pediatric population and found that males and females were equally likely to be infected, but they did not specify the effect of sex on the manifestation of symptoms [61]. Our data suggest that, unlike in adults, male sex in children may be associated with the absence of symptoms. 

The characteristics of COVID-19 did not differ greatly between patients <10 years (including neonates and infants) and ≥10 years. Elevated serum creatinine levels were present at higher rates in older patients with symptoms. It is worth noting that typical GGOs were present at higher frequencies within asymptomatic older patients (83.3%) and were only present in 20.8% of younger asymptomatic patients. Liu et al. evaluated CT imaging as a diagnostic tool in children when nucleic acid tests were unavailable and found less lung involvement in children. However, these results indicate that chest CT may be more specific within an older pediatric population, which warrants further study. 

Our data confirms previous findings that demonstrated milder infection in the pediatric population. No deaths were reported, and most cases recovered fully. Asymptomatic patients rarely received treatment, and symptomatic patients typically recovered well with antibiotics, antivirals, and oxygen therapy. For both asymptomatic and symptomatic populations, ICU admission occurred at similar rates. Only one ARDS was observed, which was consistent with previous research that complication rate of children was <2% as compared to 5% in adults [11,30,54]. The most common symptoms, when present, were fever, cough, and vomiting. Cases of neonatal COVID-19 were reported in 15 cases across six studies [14,15,18,19,37,39], and prognoses and symptoms were similar to other age groups, with six neonatal infections being asymptomatic. 

There is still not enough information about the clinical features, laboratory, and radiologic findings of children, infants, and neonates COVID-19 infections. Fortunately, the pediatric population is deemed to have milder symptoms and better prognosis than adults. Thus, clinicians should be alert to asymptomatic SARS-CoV-2 infected children. Children cannot stay at home forever, and they need more definite preventive and treatment measures than just hand hygiene and supportive care. Therefore, continuous updates on the susceptibility and mechanisms on childhood COVID-19 infection are important. 

Our study has several limitations. First, blood laboratory samples and CT were taken at different time points of infection for different patients. It is possible that as disease progressed from the contraction of illness to the onset of symptoms, laboratory characteristics and radiological features could have changed among cases. Second, included articles were not prospective, which would have helped in identifying risk factors predisposing certain children to symptomatic infection. Third, most articles originated from China, as studies from other countries with regards to children were rare. Furthermore, we found low numbers of cases within some categories of age and symptoms. For example, the subset of symptomatic patients ≥10 years with reported GGOs included only three cases, which could have interfered with statistical reliability. Fourth, multisystem inflammatory syndrome, a severe pediatric condition associated with COVID-19, was not specified during data extraction. Although the condition is rare, with a total of 570 reported to the CDC as of July 2020, it is possible that signs and symptoms of multisystem inflammatory syndrome in one or more samples included in our analysis could have altered our results [62]. Fifth, although this study aggregates evidence sufficiently to achieve a power of 0.75–0.85 for comparisons of asymptomatic and symptomatic or younger and older children, some subgroup comparisons performed using Fisher’s exact test were unable to achieve this level of power, ranging from as low as 0.15 for GGO comparisons in asymptomatic vs. symptomatic children below 10 years to as high as 0.90 for GGO comparisons in above vs. below age 10 in asymptomatic children. Finally, we were unable to perform contact-tracing on our cases to evaluate the transmissibility of the symptomatic and asymptomatic cases. However, the strength of this systematic review is that it is the first, to our knowledge, to aggregate evidence of symptomatic and asymptomatic SARS-CoV-2 infection in children of varying ages. 

## 5. Conclusions

Our systematic review summarizes the clinical features of 158 pediatric COVID-19 patients stratified by both the presence of symptoms and by groups above and below 10 years old. We report no mortalities, and most cases recovered. However, males were more likely to have asymptomatic infection, and abnormal lab findings better distinguished symptomatic from asymptomatic pediatric COVID-19. The clinical features of the older and younger age groups were similar, but typical GGOs were present more often in older asymptomatic infections. Furthermore, prospective studies could reveal predisposing factors of symptomatic or asymptomatic infection and guide prevention efforts.

## Figures and Tables

**Table 1:** Baseline demographic, clinical characteristics, radiologic and laboratory findings according to symptoms and age group in children ^1^.

	Symptoms			Age			
Variables	Asymptomatic (*n* = 51)	Symptomatic (*n* = 107)	*p* Value	<10 Years (*n* = 116)	≥10 Years (*n* = 40)	*p* Value	Total (*n* = 158)
**Age (years)**	5.84 ± 4.91	5.39 ± 5.20	0.606	3.09 ± 3.21	12.60 ± 1.97	**0.000**	5.53 ± 5.08
Male	31/47 (66.0%)	50/103 (48.5%)	0.054	56/109 (51.4%)	24/40 (60.0%)	0.362	81/150 (54.0%)
Female	16/47 (34.0%)	53/103 (51.5%)	0.054	47/97 (48.5%)	11/27 (40.7%)	0.519	69/150 (46.0%)
**Country (location)**			**0.000**			**0.007**	
China	22/24 (91.7%)	68/96 (70.8%)	-	75/91 (82.4%)	14/28 (50.0%)	-	90/120 (75.0%)
Europe	-	20/96 (20.8%)	-	10/91 (11.0%)	10/28 (35.7%)	-	20/120 (16.7%)
Malaysia	1/24 (4.2%)	3/96 (3.1%)	-	3/91 (3.3%)	1/28 (3.6%)	-	4/120 (3.3%)
United States	-	3/96 (3.1%)	-	1/91 (1.1%)	2/28 (7.1%)	-	3/120 (2.5%)
Republic of Korea	-	1/96 (1.0%)	-	-	1/28 (3.6%)	-	1/120 (0.8%)
Vietnam	-	1/96 (1.0%)	-	1/91 (1.1%)	-	-	1/120 (0.8%)
Singapore	1/24 (4.2%)	-	-	1/91 (1.1%)	-	-	1/120 (0.8%)
Asymptomatic during follow-up	23/24 (95.8%)	-	-	14/14 (100.0%)	8/9 (88.9%)	0.391	23/24 (95.8%)
Radiology abnormal	15/31 (48.4%)	44/69 (63.8%)	0.188	41/75 (54.7%)	17/23 (73.9%)	0.145	59/100 (59.0%)
Typical ground-glass opacities with patchy shadows	4/31 (12.9%)	15/69 (21.7%)	0.411	13/75 (17.3%)	6/23 (26.1%)	0.374	19/100 (19.0%)
Typical ground-glass opacities with consolidation	2/31 (6.5%)	0/69 (0.0%)	0.094	2/75 (2.7%)	0/23 (0.0%)	1.000	2/100 (2.0%)
Typical ground-glass opacities only	3/31 (9.7%)	11/68 (16.2%)	0.539	8/75 (10.7%)	6/22 (27.3%)	0.080	14/99 (14.1%)
**Typical ground-glass opacities**	10/31 (32.3%)	27/69 (39.1%)	0.655	24/75 (32.0%)	13/23 (56.5%)	**0.049**	37/100 (37.0%)
Consolidation	0/31 (0.0%)	3/69 (4.3%)	0.550	2/75 (2.7%)	1/23 (4.3%)	1.000	3/100 (3.0%)
Signs of pneumonia	14/31 (45.2%)	43/69 (62.3%)	0.129	39/75 (52.0%)	17/23 (73.9%)	0.091	57/100 (57.0%)
Bronchitis	1/31 (3.2%)	1/69 (1.4%)	1.000	2/75 (2.7%)	0/23 (0.0%)	1.000	2/100 (2.0%)
Laboratory abnormal	17/31 (54.8%)	61/93 (65.6%)	0.104	63/95 (66.3%)	14/31 (45.2%)	0.055	78/128 (60.9%)
WBC count, high	2/35 (5.7%)	11/93 (11.8%)	0.356	12/95 (12.6%)	1/31 (3.2%)	0.184	13/128 (10.2%)
low	1/35 (2.9%)	13/93 (14.0%)	0.110	9/95 (9.5%)	5/31 (16.1%)	0.330	14/128 (10.9%)
Neutrophil count, high	0/33 (0.0%)	5/86 (5.8%)	0.320	3/88 (3.4%)	2/29 (6.9%)	0.596	5/119 (4.2%)
low	2/33 (6.1%)	15/86 (17.4%)	0.148	13/88 (14.8%)	3/29 (10.3%)	0.758	17/119 (14.3%)
**Lymphocyte count, high**	0/35 (0.0%)	13/93 (14.0%)	**0.019**	11/95 (11.6%)	1/31 (3.2%)	0.291	13/128 (10.2%)
low	1/35 (2.9%)	10/93 (10.8%)	0.185	10/95 (10.5%)	1/31 (3.2%)	0.291	11/128 (8.6%)
Platelet count, high	0/33 (0.0%)	5/86 (5.8%)	0.320	4/88 (4.5%)	1/29 (3.4%)	1.000	5/119 (4.2%)
low	2/33 (6.1%)	2/86 (2.3%)	0.575	3/88 (3.4%)	1/29 (3.4%)	1.000	4/119 (3.4%)
Creatinine level, high	0/31 (0.0%)	2/83 (2.4%)	0.601	0/83 (0.0%)	2/29 (6.9%)	0.065	2/114 (1.8%)
low	0/31 (0.0%)	10/83 (12.0%)	0.060	10/83 (12.0%)	0/29 (0.0%)	0.061	10/114 (8.8%)
LDH level, high	1/33 (3.0%)	7/88 (8.0%)	0.444	7/88 (8.0%)	1/31 (3.2%)	0.454	8/121 (6.6%)
**CRP, high**	2/35 (5.7%)	21/92 (22.8%)	**0.037**	17/94 (18.1%)	6/31 (19.4%)	1.000	23/127 (18.1%)
High ALT level, high	3/33 (9.1%)	7/90 (7.8%)	1.000	9/90 (10.0%)	1/31 (3.2%)	0.295	10/123 (8.1%)
low	0/33 (0.0%)	3/90 (3.3%)	0.563	1/90 (1.1%)	2/31 (6.5%)	0.161	3/123 (2.4%)
Procalcitonin level, high	1/8 (12.5%)	12/22 (54.5%)	0.092	9/20 (45.0%)	4/10 (40.0%)	1.000	13/30 (43.3%)
D-dimer level, high	2/7 (28.6%)	9/29 (31.0%)	1.000	10/28 (35.7%)	1/8 (12.5%)	0.388	11/36 (30.6%)
CK-MB level, high	7/11 (63.6%)	8/22 (36.4%)	0.266	14/28 (50.0%)	1/5 (20.0%)	0.346	15/33 (45.5%)

^1^ Data are mean ± SD or number (percentage) of patients. Different denominators are due to unavailable results, or because the test is not done. Values in bold indicate statistically significant results. Abbreviations: WBC (white blood cells), LDH (lactose dehydrogenase), CRP (C-reactive protein), ALT (alanine aminotransferase), CK-MB (creatine kinase MB fraction).

**Table 2:** Baseline demographic, clinical characteristics, radiologic, and laboratory findings in children according to age ^1^.

	<10 Years (*n* = 116)			≥10 Years (*n* = 40)		
Variables	Asymptomatic (*n* = 35)	Symptomatic (*n* = 81)	*p* Value	Asymptomatic (*n* = 15)	Symptomatic (*n* = 25)	*p* Value
Age (years)	3.26 ± 3.24	3.02 ± 3.22	0.722	11.87 ± 1.77	13.04 ± 1.99	0.068
Male	19/31 (61.3%)	37/78 (47.4%)	0.210	11/15 (73.3%)	13/25 (52.0%)	0.318
Female	11/30 (36.7%)	36/68 (52.9%)	0.188	3/14 (21.4%)	8/14 (57.1%)	0.120
Asymptomatic during follow-up	14/14 (100.0%)	-	-	8/9 (88.9%)	-	-
Radiology abnormal	10/24 (41.7%)	31/51 (60.8%)	0.142	5/6 (83.3%)	12/17 (70.6%)	1.000
Typical ground-glass opacities with patchy shadows	2/24 (8.3%)	11/51 (21.6%)	0.203	2/6 (33.3%)	4/17 (23.5%)	0.632
Typical ground-glass opacities with consolidation	2/24 (8.3%)	0/51 (0.0%)	0.099	0/6 (0.0%)	0/17 (0.0%)	-
**Typical ground-glass opacities only**	0/24 (0.0%)	8/51 (15.7%)	**0.049**	3/6 (50.0%)	3/16 (18.8%)	0.283
Typical ground-glass opacities	5/24 (20.8%)	19/51 (37.3%)	0.191	5/6 (83.3%)	8/17 (47.1%)	0.179
Consolidation	0/24 (0.0%)	2/51 (3.9%)	0.559	0/6 (0.0%)	1/17 (5.9%)	1.000
Signs of pneumonia	9/24 (37.5%)	30/51 (58.8%)	0.136	5/6 (83.3%)	12/17 (70.6%)	1.000
Bronchitis	1/24 (4.2%)	1/51 (2.0%)	1.000	0/6 (0.0%)	0/17 (0.0%)	-
Laboratory abnormal	15/26 (57.7%)	48/69 (69.6%)	0.332	2/7 (28.6%)	11/14 (78.6%)	0.056
WBC count, high	2/26 (7.7%)	10/69 (14.5%)	0.502	0/8 (0.0%)	1/24 (4.2%)	1.000
low	0/26 (0.0%)	9/69 (13.0%)	0.108	1/8 (12.5%)	4/24 (16.7%)	1.000
Neutrophil count, high	0/24 (0.0%)	3/64 (4.7%)	0.559	0/8 (0.0%)	2/22 (9.1%)	1.000
low	1/24 (4.2%)	12/64 (18.8%)	0.104	1/8 (12.5%)	2/22 (9.1%)	1.000
**Lymphocyte count, high**	0/26 (0.0%)	11/69 (15.9%)	**0.032**	0/8 (0.0%)	2/24 (8.3%)	1.000
low	1/26 (3.8%)	9/69 (13.0%)	0.276	0/8 (0.0%)	1/24 (4.2%)	1.000
Platelet count, high	0/24 (0.0%)	4/64 (6.3%)	0.333	0/8 (0.0%)	1/22 (4.5%)	1.000
low	2/24 (8.3%)	1/64 (1.6%)	0.179	0/8 (0.0%)	1/22 (4.5%)	1.000
Creatinine level, high	0/22 (0.0%)	0/61 (0.0%)	-	0/8 (0.0%)	2/22 (9.1%)	1.000
low	0/22 (0.0%)	10/61 (16.4%)	0.056	0/8 (0.0%)	0/22 (0.0%)	-
LDH level, high	1/24 (4.2%)	6/64 (9.4%)	0.669	0/8 (0.0%)	1/24 (4.2%)	1.000
CRP level, high	2/26 (7.7%)	15/68 (22.1%)	0.139	0/8 (0.0%)	6/24 (25.0%)	0.296
ALT level, high	2/24 (8.3%)	7/66 (10.6%)	1.000	1/8 (12.5%)	0/24 (0.0%)	0.250
low	0/24 (0.0%)	1/66 (1.5%)	1.000	0/8 (0.0%)	2/24 (8.3%)	1.000
Procalcitonin level, high	1/5 (20.0%)	8/15 (53.3%)	0.319	0/3 (0.0%)	4/7 (57.1%)	0.200
D-dimer level, high	2/5 (40.0%)	8/23 (34.8%)	1.000	0/2 (0.0%)	1/6 (16.7%)	1.000
CK-MB level, high	7/10 (70.0%)	7/18 (38.9%)	0.236	0/1 (0.0%)	1/4 (25.0%)	1.000

^1^ Data are mean ± SD or number (percentage) of patients. Different denominators are due to unavailable results, or because the test is not done. Values in bold indicate statistically significant results. Abbreviations: WBC (white blood cells), LDH (lactose dehydrogenase), CRP (C-reactive protein), ALT (alanine aminotransferase), CK-MB (creatine kinase MB fraction).

**Table 3:** Baseline demographic, clinical characteristics, radiologic, and laboratory findings in children according to the presence of symptoms ^1^.

	Asymptomatic (*n* = 50)			Symptomatic (*n* = 106)		
Variables	<10 Years (*n* = 35)	≥10 Years (*n* = 15)	*p* Value	<10 Years (*n* = 71)	≥10 Years (*n* = 14)	*p* Value
**Age (years)**	3.26 ± 3.24	11.87 ± 1.77	**0.000**	3.02 ± 3.22	13.04 ± 1.99	**0.000**
Male	19/31 (61.3%)	11/15 (73.3%)	0.520	37/78 (47.4%)	13/25 (52.0%)	0.819
Female	11/29 (37.9%)	3/10 (30.0%)	0.485	36/68 (52.9%)	8/14 (57.1%)	1.000
Asymptomatic during follow-up	14/14 (100.0%)	8/9 (88.9%)	0.391	-	-	-
Radiology abnormal	10/24 (41.7%)	5/6 (83.3%)	0.169	31/51 (60.8%)	12/17 (70.6%)	0.568
Typical ground-glass opacities with patchy shadows	2/24 (8.3%)	2/6 (33.3%)	0.169	11/51 (21.6%)	4/17 (23.5%)	1.000
Typical ground-glass opacities with consolidation	2/24 (8.3%)	0/6 (0.0%)	1.000	0/51 (0.0%)	0/17 (0.0%)	-
**Typical ground-glass opacities only**	0/24 (0.0%)	3/6 (50.0%)	**0.005**	8/51 (15.7%)	3/16 (18.8%)	0.716
**Typical ground-glass opacities**	5/24 (20.8%)	5/6 (83.3%)	**0.009**	19/51 (37.3%)	8/17 (47.1%)	0.570
Consolidation	0/24 (0.0%)	0/4 (0.0%)	-	2/51 (3.9%)	1/17 (5.9%)	1.000
Signs of pneumonia	9/24 (37.5%)	5/6 (83.3%)	0.072	30/51 (58.8%)	12/17 (70.6%)	0.565
Bronchitis	1/24 (4.2%)	0/6 (0.0%)	1.000	1/51 (2.0%)	0/17 (0.0%)	1.000
Laboratory abnormal	15/24 (62.5%)	2/8 (25.0%)	0.225	48/69 (69.6%)	12/23 (52.2%)	0.206
WBC count, high	2/26 (7.7%)	0/8 (0.0%)	1.000	10/69 (14.5%)	1/23 (4.3%)	0.280
low	0/26 (0.0%)	1/8 (12.5%)	0.235	9/69 (13.0%)	4/23 (17.4%)	0.730
Neutrophil count, high	0/24 (0.0%)	0/8 (0.0%)	-	3/64 (4.7%)	2/21 (9.5%)	0.593
low	1/24 (4.2%)	1/8 (12.5%)	0.444	12/64 (18.8%)	2/21 (9.5%)	0.501
Lymphocyte count, high	0/26 (0.0%)	0/8 (0.0%)	-	11/69 (15.9%)	1/23 (4.3%)	0.282
low	1/26 (3.8%)	0/8 (0.0%)	1.000	9/69 (13.0%)	1/23 (4.3%)	0.442
Platelet count, high	0/24 (0.0%)	0/8 (0.0%)	-	4/64 (6.3%)	1/21 (4.8%)	1.000
low	2/24 (8.3%)	0/8 (0.0%)	1.000	1/64 (1.6%)	1/21 (4.8%)	0.435
Creatinine level, high	0/22 (0.0%)	0/8 (0.0%)	-	0/61 (0.0%)	2/21 (9.5%)	0.063
low	0/22 (0.0%)	0/8 (0.0%)	-	10/61 (16.4%)	0/21 (0.0%)	0.058
LDH level, high	1/24 (4.2%)	0/8 (0.0%)	1.000	6/64 (9.4%)	1/23 (4.3%)	0.670
CRP level, high	2/26 (7.7%)	0/8 (0.0%)	1.000	15/68 (22.1%)	6/23 (26.1%)	0.776
ALT level, high	2/24 (8.3%)	1/8 (12.5%)	1.000	7/66 (10.6%)	0/23 (0.0%)	0.183
low	0/24 (0.0%)	0/8 (0.0%)	-	1/66 (1.5%)	2/23 (8.7%)	0.163
Procalcitonin level, high	1/5 (20.0%)	0/3 (0.0%)	1.000	8/16 (50.0%)	4/7 (57.1%)	1.000
D-dimer level, high	2/5 (40.0%)	0/2 (0.0%)	1.000	8/24 (33.3%)	1/6 (16.7%)	0.637
CK-MB level, high	7/10 (70.0%)	-	-	7/18 (38.9%)	1/4 (25.0)	1.000

^1^ Data are mean ± SD or number (percentage) of patients. Different denominators are due to unavailable results, or because the test is not done. Values in bold indicate statistically significant results. Abbreviations: WBC (white blood cells), LDH (lactose dehydrogenase), CRP (C-reactive protein), ALT (alanine aminotransferase), CK-MB (creatine kinase MB fraction).

## Supplementary Materials

The following are available online at https://www.mdpi.com/1648-9144/56/9/474/s1, Table S1: Summary profiles of asymptomatic patients, Table S2: Summary profiles of symptomatic patients.
