# Supplementary material for: Clinical Characteristics of Asymptomatic and Symptomatic Pediatric Coronavirus Disease 2019 (COVID-19): A Systematic Review
Source: Medicina (Kaunas). 2020 Sep 15;56(9):474. doi: 10.3390/medicina56090474 (PMC7558873; doi:10.3390/medicina56090474)
Supplement: Supplementary file 1 [file medicina-56-00474-s001.pdf]

Online-Only Supplement

Clinical Characteristics of Asymptomatic and Symptomatic Pediatric Coronavirus Disease 2019 (COVID-19): A Systematic Review

Table S1. Summary profiles of asymptomatic patients.
Table S2. Summary profiles of symptomatic patients.
References

Table S1. Summary profiles of asymptomatic patients.

Author (year)	Age / Sex	Country	Presenting symptoms	Lab findings				Image findings	Remarkable past history	ICU admission	ARDS	Treatment	Outcome	Survival
			Others	CBC	CRP	Chemistry	Others							
**1. Li et al. (2020)[1]**	1Y5M/M		-		9.4mg/L			Four days after admission, patchy ground-glass opacification of the right upper lobe. Five days subsequently, during antiviral treatment, normalized.		Yes		Antiviral, anti-infective therapy, immunoglobulin therapy, interferon, Lianhua qingwen granules	Remain in hospital for observation; 24 days at time of writing	Alive
	10M/F		-	WBC 14.8*10⁹/L				Not performed.		Yes		Montelukast Na chewable tablets, immunoglobulin therapy	Discharged home	Alive
	4Y/M		-					Two days after admission, patchy ground-glass opacification of the left lower lobe. Five days subsequently, during symptomatic and supportive care, normalized.		Yes		Montelukast Na chewable tablets, immunoglobulin therapy	Discharged home	Alive
	6Y/M		-					Not performed.		?		Interferon, Montelukast Na chewable tablets, immunoglobulin therapy	Remain in hospital for observation; 13 days at time of writing	Alive
**2. Wang et al.**	36h/M	China	-					CT: high-density nodular shadow under	-	-	-	No special treatment was	Discharged	Alive

**(2020)[2]**						the pleura of the posterior segment of the upper lobe of the right lung				given		
**3. Cao et al. (2020)[3]**	17d/M	China	-	-		-	-	-	-	-	-	-
**4. Xu et al. (2020)[4]**	13Y/M	China	-	Decreased leukocytes, decreased neutrophils						A-interferon oral spray		Alive
**5. Ma et al. (2020)[5]**	3Y/M	China	-								Improved	Alive
	2Y/F	China	-								Improved	Alive
	11M/M	China	-								Improved	Alive
	9Y/F	China	-								Improved	Alive
**6. Zeng et al. (2020)[6]**	2d/M	China	-	Leukocytosis, thrombocytopenia	*Enterobacter* agglomerates–positive blood culture, coagulopathy	CT: pneumonia	Neonatal respiratory distress syndrome, pneumonia				Negative	Alive
**7. Wei et al. (2020)[7]**	8M/F	China	-					No				
**8. See et al. (2020)[8]**	9Y/M	Malaysia (from china	-			X-ray: Rt perihilar opacities	None	No	No	None		Alive

		)											
**9. Poli et al. (2020)[9]**	1M/M	Italy	-					Cystic fibrosis	No	No	None		Alive
**10. Yang et al. (2020)[10]**	38w +2	China	-						No			Discharged	Alive
	36w +2	China	-	Normal CBC	Normal	High myocardial globin, high CK-MB, high D-dimer (746)	X-ray: (premature) bilateral GO, granular high-density shadows		Yes			Discharged	Alive
	36w +2	China	-						No			Discharged	Alive
	38w	China	Eyelid dermatitis	Normal CBC	Normal	High AST (107), high myocardial globin, high CK-MB, high D-dimer (429)			Yes		No oxygen, no antiviral	Discharged	Alive
**11. Tan et al. (2020)[11]**	8Y10M/M	China	-	Normal CBC	Normal	Normal		None			No oxygen, no antiviral	Discharged	Alive
	8Y10M/F	China	Moan	Normal CBC	Normal	Normal		None			No oxygen, no antiviral	Discharged	Alive
**12. Zhang et al. (2020)[12]**	14Y/M	China	Moan	Normal				None	No	No	Lopinavir, ritonavir, arbidol, oxygen inhalation (21%, room air)	Discharged	Alive
	13Y/M	China	-					None	No	No	Oseltamivir, Lopinavir, ritonavir, arbidol, oxygen inhalation (21%, room air)	Discharged	Alive

**13. Hu et al. (2020)[13]**	10Y/F	China	-	Normal	-		CT typical ground-glass/patchy shadows						
	5Y/M	China	-	Abnormal lymphocyte	Abnormal procalcitonin, D-dimer		-						
	8Y/F	China	-		Abnormal procalcitonin level		-						
	14Y/F	China	-	-	-		-						
	6Y/M	China	-	Abnormal lymphocyte	Abnormal LDH, ALT		-						
	15Y/M	China	-		Abnormal ALT		CT typical ground-glass/patchy shadows						
**14. Chan et al. (2020)[14]**	10Y/M	China	-		Elevated alkaline phosphatase		CT ground glass	None	No	No	Supportive care	Stable	Alive
**15. Kam et al. (2020)[15]**	6M/M	Singapore	-	Neutropenia d8 (absolute neutrophil count 0.9 * 10^9/L)	-	Persistent positive PCR for 16 days	Not performed	-	No	No	None	Improved	Alive
**16. Tang et al. (2020)[16]**	10Y/M		-	Normal	Normal		-						
	10Y/M	China										Resolved	Alive
**17. Qian et al. (2020)[17]**	1Y/F		-	-	-		-						
**18. Lu et**	-/F		-	Normal	Normal		Ground glass and						

**al. (2020)[18]**					patchy opacities in both lungs
	-/M	-	Normal	Normal	-
**19. Pan et al. (2020)[19]**	3Y/M	-	Normal	Normal	-
**20. Tong et al. (2020)[20]**	12Y/M	-	-	-	-
**21. Gautret et al. (2020)[21]**	10Y/M	-	-	-	-
	12Y/F	-	-	-	-
	14Y/F	-	-	-	-
	10Y/M	-	-	-	-
**22. Su et al. (2020)[22]**	2Y/F	-	-	Increased CK-MB	-
	3Y/F	-	Thrombopenia	Increased CK-MB	Pneumonia
	5Y/F	-	-	Increased CK-MB	-
	1Y/M	-	-	Increased CK-MB	Ground glass opacities, consolidation
	1Y/M	-	-	Increased CK-MB	-
	9Y/F	-	-	-	Bronchitis

**23. Yao et al. (2020)[23]**	4Y/M		-	Normal		Normal		-				
	1Y/F		-	Normal		Normal		-				
24. Lan et al. (2020)[24]	7Y/M	China		Normal	Normal		Normal	Normal	No	No	Discharged	Alive
	7Y/F	China		Normal	Normal	High ALT (520), high AST (439)	Normal	Ground glass opacities, consolidation	No	No	Discharged	Alive
	12Y/F	China		Normal	Normal		Normal	Ground glass opacities	No	No	Discharged	Alive
	13Y/M	China		Normal	Normal		Normal	Ground glass opacities	No	No	Discharged	Alive

*Sun et al. (2020). Treatment in () means other treatments.

†Lou et al. did state that two of the three patients presented nasal congestion and rhinitis, but did not specify which.

Abbreviation: ALT(alanine transferase), ARDS(acute respiratory distress syndrome), AST(aspartate transferase), CBC(complete blood count), CK(creatine kinase), CK-MB(creatine kinase-myocardial band), Cr(creatinine), CRP(C-reactive protein), EEG(electroencephalography), ESR(Erythrocyte sedimentation ratio), GI(gastrointestinal), Hb(hemoglobin), ICU(intensive care unit), IL(interleukin), LDH(lactate dehydrogenase), PCR(polymerase chain reaction), TNF(tumor necrotizing factor), WBC(white blood cell).

Table S2. Summary profiles of symptomatic patients.

Author (year)	Age/Sex	Country	Presenting symptoms				Lab findings				Image findings	Remarkable past history	ICU admission	ARDS	Treatment	Outcome	Survival
			Fever	Respiratory	GI	Others	CBC	CRP	Chemistry	Others							
**1. Li et al. (2020)[1]**	3 Y/M		Fever	Cough, Sputum, Runny nose	-	-	WBC 15.0*10^9/L				Nine days after onset of symptoms, patchy ground-glass opacification of the left lower lobe. Seven days subsequently, during antiviral treatment, normalized.		?		Antiviral, anti-infective therapy, immunoglobulin therapy	Discharged home	Alive
**2. Liu et al. (2020)[25]**	3 Y/F	China	High fever (>39 ℃)	Cough, Tachypnea, Pharyngeal congestion, Swollen tonsils	Vomiting	-	Low WBC, low neutrophil count, low lymphocyte count, low Hb	High CRP	High D-dimer, high AST, low Cr, high LDH, high Mg, low bicarbonate		Patchy ground-glass opacities in both lungs		Yes		Ribavirin, Oseltamivir, Glucocorticoids, Supplemental oxygen, Intravenous immune globulin		
	7 Y/F	China	High fever (>40 ℃)	Cough, Pharyngeal congestion, Swollen tonsils	-	-	Low lymphocyte count	High CRP	Low Cr, low bicarbonate		NA		No		Oseltamivir		

3 Y/F	China	High fever (>41 ℃)	Cough, Pharyngeal congestion, Swollen tonsils	Vomiting	-	Low lymphocyte count, high ESR	High CRP	High D-dimer, high AST, low Cr, high LDH, low Na, low chlorine, high Mg, low bicarbonate	Patchy shadows in both lungs	No	Oseltamivir, Glucocorticoids
1 Y/M	China	High fever (>42 ℃)	Cough, Wheeze, Pharyngeal congestion, Swollen tonsils	-	-	Low WBC, low neutrophil count, low lymphocyte count, high AST, low Cr, low bicarbonate	High CRP		Patchy shadows in both lungs	No	Oseltamivir, Glucocorticoids
3 Y/F	China	High fever (>43 ℃)	Cough, Wheeze, Swollen tonsils	Vomiting	-	Low WBC, low lymphocyte count, high ESR	High CRP	High D-dimer, high ALT, high AST, low Cr, low Na, low bicarbonate	Patchy shadows in both lungs	No	Oseltamivir, Glucocorticoids
4 Y/M	China	High fever (>44 ℃)	Cough, Pharyngeal congestion, Swollen	Vomiting	-	Low WBC, low neutrophil count, low lymphocyte count		Low Cr, low Na, low bicarbonate	Normal	No	Ribavirin, Oseltamivir

			tonsils											
**3. Sun et al. (2020)[26]***	8 Y/M	Fever	Cough, Expectoration, Polypnea	-	-	Low leukocytes, low neutrophils, low lymphocytes, low Hb	High CRP	High procalcitonin, high LDH, high ALT	Bilateral pneumonia, Multiple patch-like shadows, GGO, "white lung" appearance	Acute lymphocytic leukemia (infection with Influenza A virus)	Yes	Oxygen therapy, Mechanical Ventilation, Antibiotic treatment, Antiviral treatment, Glucocorticoids, Intravenous immunoglobulin therapy, (Traditional Chinese medicine) *	Remained in ICU	Alive
	10 M /F	-	Cough, Expectoration, Polypnea	Constipation, Nausea, Vomiting	-	High leukocytes, high neutrophils, low thrombocyte, low Hb	High CRP	High procalcitonin, high LDH, high ALT, high CK, high D-dimer	Bilateral pneumonia, Multiple patch-like shadows, pleural effusion, GGO	Lacrimal sac dredge	Yes	Oxygen therapy, Antibiotic treatment, Antiviral treatment, Glucocorticoids, Intravenous immunoglobulin therapy, (Enterostomy, hemopurification, transfusions of red blood cell, plasma and thrombocyte)	Remained in ICU	Alive
	1 Y 1 M / M	Fever	Polypnea	Diarrhea, Nausea, Vomiting	-	Low Hb		Low creatine, high D-dimer	Bilateral pneumonia, Multiple patch-like shadows, GGO		Yes	Oxygen therapy, Mechanical Ventilation, Antibiotic treatment, Antiviral treatment, Glucocorticoids,	Discharged	Alive

										(Plasmapheresis)		
2M/M	-	Cough, Expectoration, Polypnea	Nausea, Vomiting	-	High lymphocytes, high thrombocyte		High procalcitonin, high LDH, high ALT, low Cr	Unilateral pneumonia, Multiple patch-like shadows		Yes	Oxygen therapy, Antiviral treatment	Discharged	Alive
2Y1M/M	Fever	Cough, Polypnea	Diarrhea, Nausea, Vomiting	-		High CRP	High procalcitonin, high LDH, low AST, high ALT, high Cr kinase, low Cr	Bilateral pneumonia, Multiple patch-like	Pharyngitis	Yes	Antibiotic treatment, Antiviral treatment	Discharged	Alive
15Y/F	Fever	Cough, Polypnea	Diarrhea	-	Mildly High leukocytes, mildly high neutrophils, mildly high lymphocytes, high thrombocyte		High LDH, low AST, low ALT, high Cr	Bilateral pneumonia, Multiple patch-like shadows, GGO		Yes	Antibiotic treatment, Antiviral treatment, Glucocorticoids, Intravenous immunoglobulin therapy, (Traditional Chinese medicine)	Discharged	Alive
13Y11M/M	Fever	Cough, Polypnea	-	-	Mildly high Hb	High CRP	High procalcitonin, low AST	Bilateral pneumonia, GGO		Yes	Oxygen therapy, Antiviral treatment, Glucocorticoids, Intravenous immunoglobulin therapy, (Traditional Chinese medicine)	Discharged	Alive

	13 Y 5 M / M		Fever	Expectoration, Polypnea	-	-			Low AST, low ALT, high Cr	Unilateral pneumonia, Multiple mottling, GGO		Yes		Oxygen therapy, Antiviral treatment, (Traditional Chinese medicine)	Remained in ICU	Alive
**4. Ji et al. (2020)[27]**	15 Y/ M	China	Fever (37.9 ℃)	Pharyngeal congestion	-	-	WBC 11.82*10⁹/L (67.3% neutrophils, 25.7% lymphocytes)	34.64 mg/ L		Normal	-	-	-	Symptomatic treatment was given	Symptoms disappeared after treatment for two days	Alive
	9 Y/ M	China	-	Small amount of sputum	Mild diarrhea	-	WBC 6.6*10⁹/L (34.1% neutrophils, 52% lymphocytes)	3.49 mg/ L		Normal	-	-	-	Oral probiotic	Symptoms disappeared after treatment for two days	Alive
**5. Park et al. (2020)[28]**	10 Y/ F	Korea	Fever	Sputum	-	-	WBC 4,080/μL (37.3% lymphocytes), Hb 13.5g/dl, platelet count 251,000/μL	<0.4 mg/ dl		CT: Patchy or nodular consolidations with peripheral ground glass opacities in subpleural areas of the right lower lobe	-	-	-	-	-	-
**6. Cao et al. (2020)[3]**	3 M /F	China	Fever	-	-	-	WBC 9690/mm³ (45% neutrophils, 44% lymphocytes)			CT: mildly increased infiltrates at bilateral lung				Supportive treatment	Discharged	Alive

	7 Y/M	China	Fever	-	-	-	-		-	-	-	-	-	-	-
	-	China	-	Runny nose	Vomiting	-	WBC 7660/mm³ (15% neutrophils, 73% lymphocytes)		CT: mildly increased bilateral linear opacities					-	-
	30 h/-	China	-	Respiratory distress without fever	-	-	-		-	-	-	-	-	-	-
	1 Y/M	China	Fever	Respiratory distress	Diarrhea, Vomiting	-	-		CT: showed pneumonia	-	Yes	-	Assisted ventilation, continuous venovenous hemofiltration	Recovered gradually	Alive
**7. Cui et al. (2020)[29]**	55 D/F	China	-	Dry cough, Rhinorrhea	-	-	Elevated lymphocyte, platelet	Elevated AST/ALT, CK	CT: Patchy shadows and GGO in Rt. lung	-	No	No	Inhaled interferon α-1b, amoxicillin potassium clavulanate, reduced glutathione, ursodeoxycholic acid, traditional Chinese medicine lotus qingwen	Improved	Alive
**8. Spiteri et al. (2020)[30]**	4 cases ≤ 17	Europe	-	-	-	-									

	years old											
**9. Xu et al. (2020)[4]**	6 Y/M	China	Fever	Cough	Diarrhea	-	Elevated neutrophils, decreased lymphocytes, elevated ESR		Elevated procalcitonin		A-interferon oral spray, azithromycin, IVIG	Alive
	12 Y/F	China	Fever	Sore throat, Rhinorrhea	-	-	Decreased leukocytes, Decreased lymphocytes		Increased procalcitonin		A-interferon oral spray	Alive
	7 Y/F	China	Fever	Cough, Sore throat	Diarrhea	-	Decreased lymphocytes	Increased CRP	Increased procalcitonin		A-interferon oral spray	Alive
	1 Y/M	China	Fever	-	-	-	Decreased neutrophils, increased lymphocytes, increased ESR		Increased AST		A-interferon oral spray	Alive
	3 Y/M	China	-	Rhinorrhea	-	-	None				A-interferon oral spray	Alive
	15 Y/F	China	Fever	-	-	-	Decreased leukocytes, decreased neutrophils,	Increased CRP			A-interferon oral spray	Alive

							increased ESR								
	13 Y/M	China	Fever	Cough, Sore throat	-	-		Increased CRP	Increased procalcitonin				A-interferon oral spray		Alive
	2 M/F	China	-	Cough, Sore throat	-	-	Decreased neutrophil, increased lymphocytes		Increased AST/ALT, increased procalcitonin				A-interferon oral spray		Alive
	1 Y/M	China	Fever	Cough	Diarrhea	-	Not available						A-interferon oral spray		Alive
**10. Liu et al. (2020)[31]**	5 Y/F	China	Fever	Cough	-	Fatigue	Decreased leukocyte count, decreased neutrophil ratio, increased lymphocyte count, increased lymphocyte ratio			Normal	No	No	-	Stable	Alive
	11 M/M	China	Fever	Cough	-	-	Decreased neutrophil ratio, increased lymphocyte count, increased lymphocyte ratio			Single consolidation	No	No	-		Alive
	9 Y/F	China	Fever	-	-	-	Decreased lymphocyte ratio			GGO	No	No	-		Alive

	2 M/M	China	-	Cough	-	Fatigue		Increased CRP			Multiple consolidations	No	No	-		Alive
**11. Zhang et al. (2020)[32]**	9 Y/M		Fever	Nasal congestion, Sore throat, Runny nose	Gastric appetite, Nausea	Muscle pain, Headache	Mild high WBC		High CRP, low CK	High IL-6				Interferon, Chinese medicine, vitamin C, antibiotic application		
	6 Y/M		-	Cough	-	-	High Hb		Low CK					Interferon, Chinese medicine, vitamin C		
	8 Y/M		Low grade fever	Nasal congestion, Rhinorrhea	-	-	High Hb, low neutrophil ratio, high lymphocyte ratio		Low ALT, low CK					Interferon, Chinese medicine, vitamin C		
**12. Le et al. (2020)[33]**	3 M/F	Vietnam	Fever	Rhinorrhea	-	Myalgia	Mild high WBC		High CK			Yes		Azithromycin,	Discharged	
**13. Ma et al. (2020)[5]**	8 Y/F	China	Fever	-	-	-					-				Improved	Alive
	3 Y/F	China	Fever	-	-	-									Improved	Alive
**14. Lou et al. (2020)[34]†**	6 Y/F	China	Fever	Cough	-	-								Interferon-α2b		
	8 Y/F	China	Fever	-	-	-								Interferon-α2b		

	6 M/M	China	Fever	-	-	-										
**15. Li et al. (2020)[35]**	10 M/M	China	Fever	-	-	-	Increased lymphocytes	Increased CRP			CT: diffuse GGO					
**16. Dong et al. (2020)[36]**	3 Y/M	China	-	Cough with phlegm	-	-	Normal	Normal	Normal	Normal serum IgE	CT: signs of pneumonia in the left upper lobe	Allergic rhinitis Community-acquired pneumonia (CAP)		Inhalation of interferon-α Supportive care	Discharged	Alive
	2 Y 4 M/M	China	Fever (39.2 ℃)	-	-	-	Normal	Normal	Normal	Serum IgE level: 173 IU/ml	CT: bilateral pneumonia	Atopic dermatitis		Inhalation of interferon-α Supportive care	Discharged	Alive
**17. Zeng et al. (2020)[6]**	2 d/M	China	Fever	-	-	Lethargy	Normal				CT: pneumonia		Yes		Negative	Alive
	2 d/M	China	Fever	-	Vomiting	Lethargy	Leukocytosis, lymphocytopenia		Elevated CK–MB fraction		CT: pneumonia				Negative	Alive
**18. Su et al. (2020)[22]**	3 Y 7 M/F	China	Fever	-	-	-	WBC $7.55*10^9$/L (20.4% neutrophils, 73.6% lymphocytes)	0.35 mg/L			Bronchitis				Discharged	Alive
	8 Y	China	Fever	-	-	-	WBC $3.78*10^9$/L	0.19 mg/			Negative				Discharged	Alive

	1 M/F						(38.7% neutrophils, 43.9% lymphocytes)	L						
	5 Y 7 M/F	China	-	Mild, dry cough	-	-	WBC $3.69*10^9$/L (33.8% neutrophils, 53.3% lymphocytes)	0.12 mg/L		Negative			Recover	Alive
**19. Cai et al. (2020)[37]**	7 Y/M	China	Fever	Cough	-	-	Increased WBC, Increased neutrophil	Increased CRP	Increased CK-MB, Increased D-dimer		-	Symptomatic Treatment		
	10 Y/F	China	Fever	Sore throat, Stuffy nose	-	-						Symptomatic Treatment		
	10 Y/F	China	Fever	Cough, Sore throat, Stuffy nose	-	-		Increased CRP	Increased CK-MB	X-ray: Lt. retrocardiac opacity		Symptomatic Treatment		
	9 Y/M	China	Fever	Cough, Rhinorrhea, Sneezing, Sore throat	-	-	Decreased WBC, Decreased neutrophil	Increased CRP	Increased D-dimer	X-ray: Opacities in Rt. Lung		Symptomatic Treatment		
	7 M/F	China	-	Cough, Rhinorrhea, Sneezing, Stuffy nose	-	-			Increased CK-MB, Increased ALT, Increased AST	X-ray: Opacities in Rt. Lung		Symptomatic Treatment		
	6	China	Feve	Rhinorrh	-	-	Decreased					Symptomatic		

	Y/F		r	ea			neutrophil				Treatment, Antibiotic
	3 M/F	China	Fever	-	-	-	Increased lymphocyte, Increased PLATELET	Increased AST			Symptomatic Treatment, Antibiotic
	4 Y/F	China	-	Cough	-	-	Decreased neutrophil	Increased CK-MB	X-ray: Opacities in Rt. Lung		Symptomatic Treatment, Antibiotic
	8 Y/M	China	Fever	Sore throat	-	-	Increased WBC, Increased PLATELET	Increased CK-MB			Symptomatic Treatment, Antibiotic
	5 Y/M	China	Fever	Cough	-	-	Increased WBC				Symptomatic Treatment, Antibiotic
**20. Wei et al. (2020)[7]**	9 M/F	China	Fever	-	-	-				No	
	11 M/F	China	Fever	-	-	-				No	
	10 M/M	China	-	-	-	-				No	
	7 M/F	China	Fever	-	-	-				No	
	1 M/F	China	-	Cough, Rhinorrhea	-	-				No	
	3 M/F	China	-	Cough, Sputum	-	-				No	
	3	China	Feve	-	-	-				No	

	M/F		r														
	6 M/M	China	-	-	-	-							No				
**21. Odievre et al. (2020)[38]**	16 Y/F	France	Isolated fever	Respiratory distress syndrome, Superficial tachypnea 80/min	-	Acute chest pain, Tachycardia 140/min		355 mg/L	LDH 446 U/L, D-dimer 23,611 ng/ml	Spo2 85%, high IL-6 629 pg/ml, high TNF-α 32.5pg/ml, normal IL-1β normal	CTPA: bilateral pulmonary embolism complicating the ACS, bilateral consolidations with a halo sign on the right side	Homozygous SCD with bilateral ischemic retinopathy, exchange transfusions from 5 to 11yrs old, switched, thereafter for hydroxyurea	Yes	Yes	Non-invasive ventilation, red blood cell exchange transfusion followed by simple transfusion (hemoglobin nadir 6.4 g/dl), anticoagulation, 1 pulse of intravenous Tocilizumab (TCZ, 8 mg/kg),	Recover	Alive
**22. Wu et al. (2020)[39]**	2 Y 10 M/M	China	-	-	-	Conjunctivitis, Eyelid dermatitis	High lymphocyte (4.48*10^9/L)			High myoglobin	CT: normal		No	No	National protocol in China	Recover	Alive
**23. Dugue et al. (2020)[40]**	6 wks/M	USA	Fever (38.4 ℃)	Cough	-	Mild hypertension (114/57), seizure	WBC 5.07 *10^3/μL, normal differentiation		Procalcitonin(0.21ng/ml), e' normal	Rhinovirus/enterovirus PCR positive, abnormal	Brain MRI: normal	None	No	No		Discharged	Alive

										EEG							
**24. See et al. (2020)[8]**	1 Y 8 M / M	Malaysia (from china)	Mild fever	-	Diarrhea	-			Renal and liver profile normal			None	No	No	Paracetamol and Oral Rehydration Salts		Alive
	4 Y/F	Malaysia (from china)	Intermittent fever	Cough (2-4 weeks), Rhinorrhea	-	-				No blood Cx		None	No	No	Paracetamol, oral penicillin V, loratadine		Alive
	11 Y/M	Malaysia (from china)	-	Mild cough (later diagnosed as asthma)	-	-					X-ray: perihilar opacities	None	No	No	MDI salbutamol prn		Alive
**25. Yang et al. (2020)[10]**	37 w +2 / M	China	-	Vomiting	Hypoglycemia	-	Normal CBC	Normal	High myocardial globin, high CK-MB, high D-dimer (2591)				Yes			Discharged	Alive
	36 w +3	China	-	-	-	Moan, spit	Normal CBC	Normal	High procalcitonin (48hrs), high myocardial globin, high CK-MB, high D-dimer (611)		X-ray: (premature) bilateral GO, granular high-density shadows		Yes		NCPAP	Discharged	Alive
	36 w	China	-	-	-	Moan	Normal CBC	Normal	High AST (74)>48hr				Yes		NCPAP	Discharged	Alive

									normal (46), high myocardial globin, high CK-MB						
**26. Tan et al. (2020)[11]**	9Y5M/F	China	-	Cough	Abdominal pain	-	Normal CBC	Normal	High AST		CT: GGO	None	No oxygen, no antiviral	Discharged	Alive
	11Y8M/F	China	-	Cough	-	-	Normal CBC	Normal	Normal			None	No oxygen, no antiviral	Discharged	Alive
	2Y/F	China	Fever	Vomiting	-	Convulsion	Normal CBC	Normal	High AST, high CK	Mycoplasma 1:160	CT: GGO	None	No oxygen, no antiviral	Discharged	Alive
	8Y9M/M	China	Fever	-	Constipation	-	Normal CBC	Normal	Normal	Mycoplasma 1:160		None	No oxygen, no antiviral	Discharged	Alive
	12Y1M/M	China	Fever	-	-	-	Normal CBC	Normal	Normal			None	No oxygen, no antiviral	Discharged	Alive
	9Y3M/F	China	-	Cough	-	-	Normal CBC	Normal	Normal	Mycoplasma 1:80	CT: GGO, multiple nodule shadows	None	No oxygen, no antiviral	Discharged	Alive
	3	China	Feve	-	-	-	Normal	Nor	Normal		CT: GGO	None	No oxygen, no	Discharged	Alive

	Y7M/F		r				CBC	mal							antiviral		
**27. Zhang et al. (2020)[12]**	10M/F	China	Fever	Dry cough	-	Malaise	High WBC, high lymphocyte, low Hb,		High AST, low Cr, high LDH			None	No	No	Oseltamivir	Recover	Alive
28. Parri et al. (2020)[41]	4M/F	Italy	Fever	Cough, rhinorrhea, respiratory distress							CXR: normal	None			Low-flow oxygen		
	11M/M	Italy		Respiratory distress	Vomiting						CXR: normal	None			Low-flow oxygen		
	9d/F	Italy	Fever			Drowsiness, feeding difficulty		Not performed				None			High-flow oxygen		
	15Y5M/F	Italy	Fever	Cough, rhinorrhea, respiratory distress			Thrombocytopenia			frequent respiratory tract infection	CXR: patchy and ground-glass-like opacity and interstitial changes in the lungs	None			Low-flow oxygen		
	12Y6M/	Italy		Cough, respiratory distress	Nausea, vomiting						CXR: pneumonia	Autism			High-flow oxygen		

	M														
	8 d/ M	Italy	Fever			Drowsiness, feeding difficulty					None		Low-flow oxygen		
	6 Y 5 M /F	Italy	Fever			Feeding difficulty, dehydration				CXR: patchy and ground-glass-like opacity and interstitial changes in the lungs			High-flow oxygen		
	2 M / M	Italy	Fever	Cough		Feeding difficulty, skin rash				CXR: ground-glass-like opacity and interstitial changes in the lungs	Ventricular septal defect		Non-invasive ventilation		
	14 Y 5 M / M	Italy		Cough, respiratory distress		Fatigue, drowsiness, dehydration				CXR: patchy and ground-glass-like opacity and interstitial changes in the lungs	Epileptic encephalopathy (tracheotomy)		Mechanical ventilation		
29. Lee et al. (2020)[42]	15 Y/ F	USA	Fever	Cough	Abdominal pain	Decreased oral intake, fatigue	WBC 1460/uL, ANC 800cells/uL		Elevated ferritin, CRP, D-dimer, pro-BNP	Unremarkable	Familial dilated cardiomyopathy status post 3rd heart transplantation & 1st kidney		2L nasal cannula O2	Discharged	Alive

											transplan 5 mo prior, on cyclosporine, mycophenolate sodium, and low-dose prednisone, recent total lymphoid irradiation				
	13 M/M	USA		Non-productive cough, sneezing		Normal	Normal	Normal			Hypoplastic left heart syndrome, post-positive cross-match heart transplant 6 mo prior, tacrolimus, on mycophenolate mofetil, and low-dose prednisone		No change in medications (immunosuppression)	Discharged	Alive
30. Musolino, et al. (2020)[4	13 Y/M	Italy	Fever	Cough						Not performed					

3]										
	1 Y/F	Italy	Fever	Cough			High WBC	33.6 mg/L		Not performed
	15 Y/F	Italy	Fever	Cough		Chest pain				CXR: interstitial
	1 Y/F	Italy	Fever	Cough						Not performed
	15 Y/M	Italy				Seizure (not related)				Consolidation
	6 Y/M	Italy								Not performed
	16 Y/F	Italy	Fever	Cough		Chest pain, anosmia	Low WBC			Ground glass opacities
	10 Y/M	Italy	Fever		Diarrhea	Arthralgia, headache				Not performed
	12 Y/M	Italy	Fever		Diarrhea	Arthralgia, headache				Not performed
	7 Y/M	Italy	Fever			Arthralgia				Not performed

*Sun et al. (2020). Treatment in () means other treatments.
†Lou et al. did state that two of the three patients presented nasal congestion and rhinitis, but did not specify which.
Abbreviation: ALT(alanine transferase), ANC(absolute neutrophil count), ARDS(acute respiratory distress syndrome), AST(aspartate transferase), CBC(complete blood count), CK(creatine kinase), CK-MB(creatine kinase-myocardial band), Cr(creatinine), CRP(C-reactive protein), Cx(complication), EEG(electroencephalography), ESR(Erythrocyte sedimentation ratio), GI(gastrointestinal), Hb(hemoglobin), ICU(intensive care unit),

mo(month), IL(interleukin), LDH(lactate dehydrogenase), NA(not available), NCPAP(nasal continuous positive airway pressure) PCR(polymerase chain reaction), TNF(tumor necrotizing factor), WBC(white blood cell).

References

[1] Li W, Cui H, Li K, Fang Y, Li S. Chest computed tomography in children with COVID-19 respiratory infection. Pediatr Radiol. 2020;50:796-9.
[2] Wang S, Guo L, Chen L, Liu W, Cao Y, Zhang J, et al. A case report of neonatal COVID-19 infection in China. Clin Infect Dis. 2020.
[3] Cao Q, Chen YC, Chen CL, Chiu CH. SARS-CoV-2 infection in children: Transmission dynamics and clinical characteristics. J Formos Med Assoc. 2020;119:670-3.
[4] Xu Y, Li X, Zhu B, Liang H, Fang C, Gong Y, et al. Characteristics of pediatric SARS-CoV-2 infection and potential evidence for persistent fecal viral shedding. Nat Med. 2020;26:502-5.
[5] Ma X, Su L, Zhang Y, Zhang X, Gai Z, Zhang Z. Do children need a longer time to shed SARS-CoV-2 in stool than adults? Journal of Microbiology, Immunology and Infection. 2020;53:373-6.
[6] Zeng L, Xia S, Yuan W, Yan K, Xiao F, Shao J, et al. Neonatal Early-Onset Infection With SARS-CoV-2 in 33 Neonates Born to Mothers With COVID-19 in Wuhan, China. JAMA Pediatr. 2020.
[7] Wei M, Yuan J, Liu Y, Fu T, Yu X, Zhang ZJ. Novel Coronavirus Infection in Hospitalized Infants Under 1 Year of Age in China. Jama. 2020;323:1313-4.
[8] See KC, Liew SM, Ng DCE, Chew EL, Khoo EM, Sam CH, et al. COVID-19: Four Paediatric Cases in Malaysia. Int J Infect Dis. 2020;94:125-7.
[9] Poli P, Timpano S, Goffredo M, Padoan R, Badolato R. Asymptomatic case of Covid-19 in an infant with cystic fibrosis. J Cyst Fibros. 2020.
[10] Yang P, Wang X, Liu P, Wei C, He B, Zheng J, et al. Clinical characteristics and risk assessment of newborns born to mothers with COVID-19. J Clin Virol. 2020;127:104356.
[11] Tan YP, Tan BY, Pan J, Wu J, Zeng SZ, Wei HY. Epidemiologic and clinical characteristics of 10 children with coronavirus disease 2019 in Changsha, China. J Clin Virol. 2020;127:104353.
[12] Zhang B, Liu S, Dong Y, Zhang L, Zhong Q, Zou Y, et al. Positive rectal swabs in young patients recovered from coronavirus disease 2019 (COVID-19). J Infect. 2020.
[13] Hu Z, Song C, Xu C, Jin G, Chen Y, Xu X, et al. Clinical characteristics of 24 asymptomatic infections with COVID-19 screened among close contacts in Nanjing, China. Sci China Life Sci. 2020;63:706-11.
[14] Chan JF, Yuan S, Kok KH, To KK, Chu H, Yang J, et al. A familial cluster of pneumonia associated with the 2019 novel coronavirus indicating person-to-person transmission: a study of a family cluster. Lancet. 2020;395:514-23.
[15] Kam KQ, Yung CF, Cui L, Lin Tzer Pin R, Mak TM, Maiwald M, et al. A Well Infant with Coronavirus Disease 2019 (COVID-19) with High Viral Load. Clin Infect Dis. 2020.
[16] Tang A, Tong ZD, Wang HL, Dai YX, Li KF, Liu JN, et al. Detection of Novel Coronavirus by RT-PCR in Stool Specimen from Asymptomatic Child, China. Emerg Infect Dis. 2020;26:1337-9.
[17] Qian G, Yang N, Ma AHY, Wang L, Li G, Chen X, et al. A COVID-19 Transmission within a family cluster by presymptomatic infectors in China. Clin Infect Dis. 2020.
[18] Lu S, Lin J, Zhang Z, Xiao L, Jiang Z, Chen J, et al. Alert for non-respiratory symptoms of Coronavirus Disease 2019 (COVID-19) patients in epidemic period: A case report of familial cluster with three asymptomatic COVID-19 patients. J Med Virol. 2020.
[19] Pan X, Chen D, Xia Y, Wu X, Li T, Ou X, et al. Asymptomatic cases in a family cluster with SARS-CoV-2 infection. Lancet Infect Dis. 2020;20:410-1.
[20] Tong ZD, Tang A, Li KF, Li P, Wang HL, Yi JP, et al. Potential Presymptomatic Transmission of SARS-CoV-2, Zhejiang Province, China, 2020. Emerg Infect Dis. 2020;26:1052-4.
[21] Gautret P, Lagier JC, Parola P, Hoang VT, Meddeb L, Mailhe M, et al. Hydroxychloroquine and azithromycin as a treatment of COVID-19: results of an open-label non-randomized clinical trial. Int J Antimicrob Agents. 2020:105949.
[22] Su L, Ma X, Yu H, Zhang Z, Bian P, Han Y, et al. The different clinical characteristics of corona virus disease cases between children and their families in China - the character of children with COVID-19. Emerg Microbes Infect. 2020;9:707-13.
[23] Yao P, Tang P, Jiang H, Gu B, Xu P, Wang X, et al. Clusters of Human Severe Acute Respiratory Syndrome Coronavirus 2 (SARS-Cov-2) infection in a non-epidemic area, China. 2020.
[24] Lan L, Xu D, Xia C, Wang S, Yu M, Xu H. Early CT Findings of Coronavirus Disease 2019 (COVID-19) in Asymptomatic Children: A Single-Center Experience. Korean J Radiol. 2020;21:919-24.
[25] Liu W, Zhang Q, Chen J, Xiang R, Song H, Shu S, et al. Detection of Covid-19 in Children in Early January 2020 in Wuhan, China. N Engl J Med. 2020;382:1370-1.
[26] Sun D, Li H, Lu XX, Xiao H, Ren J, Zhang FR, et al. Clinical features of severe pediatric patients with coronavirus disease 2019 in Wuhan: a single center's observational study. World J Pediatr. 2020.
[27] Ji LN, Chao S, Wang YJ, Li XJ, Mu XD, Lin MG, et al. Clinical features of pediatric patients with COVID-19: a report of two family cluster cases. World J Pediatr. 2020.
[28] Park JY, Han MS, Park KU, Kim JY, Choi EH. First Pediatric Case of Coronavirus Disease 2019 in Korea. J Korean Med Sci. 2020;35:e124.

[29] Cui Y, Tian M, Huang D, Wang X, Huang Y, Fan L, et al. A 55-Day-Old Female Infant Infected With 2019 Novel Coronavirus Disease: Presenting With Pneumonia, Liver Injury, and Heart Damage. J Infect Dis. 2020;221:1775-81.
[30] Spiteri G, Fielding J, Diercke M, Campese C, Enouf V, Gaymard A, et al. First cases of coronavirus disease 2019 (COVID-19) in the WHO European Region, 24 January to 21 February 2020. Euro Surveill. 2020;25.
[31] Liu H, Liu F, Li J, Zhang T, Wang D, Lan W. Clinical and CT imaging features of the COVID-19 pneumonia: Focus on pregnant women and children. J Infect. 2020;80:e7-e13.
[32] Zhang T, Cui X, Zhao X, Wang J, Zheng J, Zheng G, et al. Detectable SARS-CoV-2 viral RNA in feces of three children during recovery period of COVID-19 pneumonia. J Med Virol. 2020;92:909-14.
[33] Le HT, Nguyen LV, Tran DM, Do HT, Tran HT, Le YT, et al. The first infant case of COVID-19 acquired from a secondary transmission in Vietnam. Lancet Child Adolesc Health. 2020;4:405-6.
[34] Lou XX, Shi CX, Zhou CC, Tian YS. Three children who recovered from novel coronavirus 2019 pneumonia. J Paediatr Child Health. 2020;56:650-1.
[35] Li D, Wang D, Dong J, Wang N, Huang H, Xu H, et al. False-Negative Results of Real-Time Reverse-Transcriptase Polymerase Chain Reaction for Severe Acute Respiratory Syndrome Coronavirus 2: Role of Deep-Learning-Based CT Diagnosis and Insights from Two Cases. Korean J Radiol: Copyright © 2020 The Korean Society of Radiology.; 2020. p. 505-8.
[36] Dong X, Cao YY, Lu XX, Zhang JJ, Du H, Yan YQ, et al. Eleven faces of coronavirus disease 2019. Allergy. 2020;75:1699-709.
[37] Cai J, Xu J, Lin D, Yang Z, Xu L, Qu Z, et al. A Case Series of children with 2019 novel coronavirus infection: clinical and epidemiological features. Clin Infect Dis. 2020.
[38] Odievre MH, de Marcellus C, Ducou Le Pointe H, Allali S, Romain AS, Youn J, et al. Dramatic improvement after tocilizumab of severe COVID-19 in a child with sickle cell disease and acute chest syndrome. Am J Hematol. 2020.
[39] Wu P, Liang L, Chen C, Nie S. A child confirmed COVID-19 with only symptoms of conjunctivitis and eyelid dermatitis. Graefes Arch Clin Exp Ophthalmol. 2020.
[40] Dugue R, Cay-Martinez KC, Thakur KT, Garcia JA, Chauhan LV, Williams SH, et al. Neurologic manifestations in an infant with COVID-19. Neurology. 2020;94:1100-2.
[41] Parri N, Lenge M, Buonsenso D, Coronavirus Infection in Pediatric Emergency Departments Research G. Children with Covid-19 in Pediatric Emergency Departments in Italy. The New England journal of medicine. 2020;383:187-90.
[42] Lee H, Mantell BS, Richmond ME, Law SP, Zuckerman WA, Addonizio LJ, et al. Varying presentations of COVID-19 in young heart transplant recipients: A case series. Pediatr Transplant. 2020;n/a:e13780.
[43] Musolino AM, Supino MC, Buonsenso D, Ferro V, Valentini P, Magistrelli A, et al. Lung Ultrasound in Children with COVID-19: Preliminary Findings. Ultrasound Med Biol. 2020;46:2094-8.
